# Supplementary figures and images for: PTM-Shepherd: Analysis and Summarization of Post-Translational and Chemical Modifications From Open Search Results
Source: Mol Cell Proteomics. 2020 Dec 11;20:100018. doi: 10.1074/mcp.TIR120.002216 (PMC7950090; doi:10.1074/mcp.TIR120.002216)

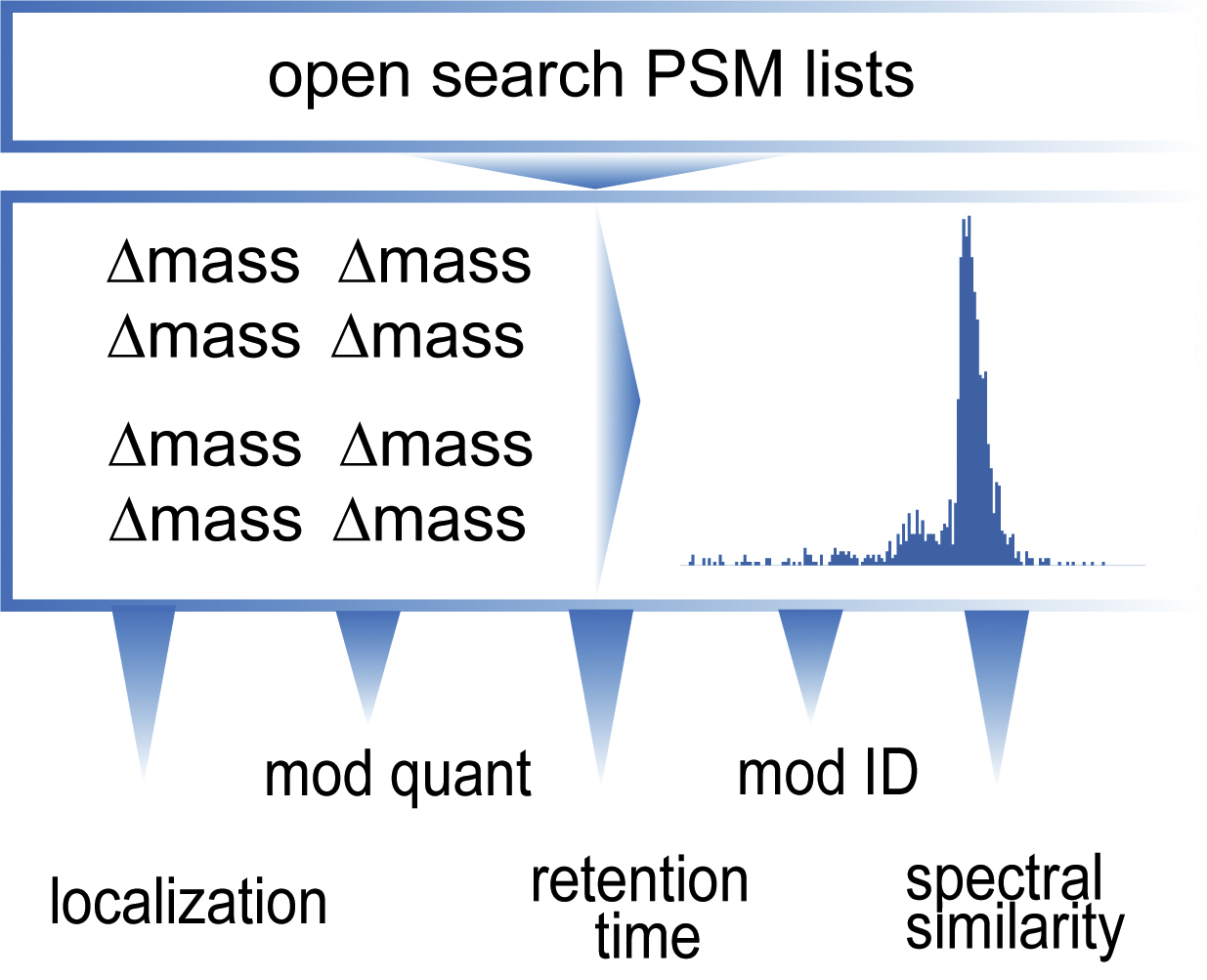

Supplement: Supplemental Material [file figs1.jpg]
